# Supplementary material for: Methylene Blue Is a Nonspecific Protein–Protein Interaction Inhibitor with Potential for Repurposing as an Antiviral for COVID-19
Source: Pharmaceuticals (Basel). 2022 May 18;15(5):621. doi: 10.3390/ph15050621 (PMC9144480; doi:10.3390/ph15050621)
Supplement: Supplementary file 1 [file pharmaceuticals-15-00621-s001.zip › pharmaceuticals-1696928-supplementary.pdf]

**SUPPLEMENTARY INFORMATION**

# Methylene Blue Is a Nonspecific Protein-Protein Interaction Inhibitor with Potential for Repurposing as an Antiviral for COVID-19

Sung-Ting Chuang[1], Henrietta Papp[2,3], Anett Kuczmog[2,3], Rebecca Eells[4], Jose M. Condor Capcha[5,6], Lina A. Shehadeh[5,6], Ferenc Jakab[2,3], and Peter Buchwald*[1,7]

[1]Diabetes Research Institute, [5]Division of Cardiology, [6]Interdisciplinary Stem Cell Institute, and [7]Department of Molecular and Cellular Pharmacology, Miller School of Medicine, University of Miami, Miami, Florida, USA

[2]National Laboratory of Virology and [3]Institute of Biology, Faculty of Sciences, University of Pécs, Pécs, Hungary

[4]Reaction Biology, Malvern, PA, USA

## Supplementary Figures

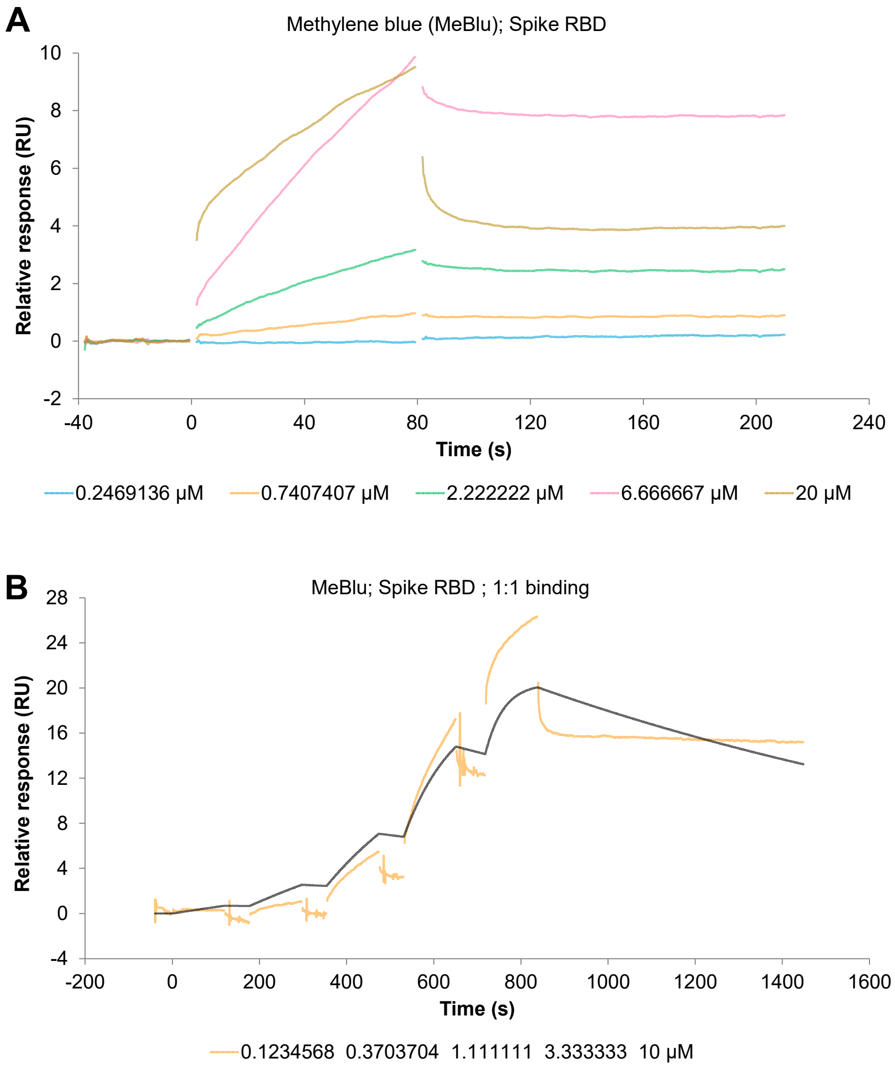


**Figure S1. Binding of MeBlu to SARS-CoV-2 spike RBD as assessed via surface plasmon resonance (SPR).** Multi- (**A**) and single- (**B**) cycle kinetics were used to measure binding to the SARS-CoV-2 spike RBD using a Biacore 8K instrument with a series S CM5 sensor chip (Cytiva) as described in the Methods. Initially a multi-cycle kinetic measurement was performed, however, the signals clearly did not return to baseline over the dissociation period and a regeneration condition was not established to ensure removal of all previously bound molecule before the injection of the next concentration. As a result, there were a decreasing number of binding sites available over the course of the measurement as sites became blocked by the preceding concentration(s), which meant this approach was not appropriate to use for a quantitative analysis even though qualitatively it was clear that binding was occurring. To deal with the apparent slow dissociation (signal not returning to baseline between injections) a single cycle kinetic approach was used instead. Data indicate low-micromolar binding; however, the binding behavior could

not be described well with a 1:1 binding interactions, thus preventing a quantitative analysis to determine the on/off-rate and/or KD values. Figure B shows an example of this deviation from 1:1 binding where the colored (yellow) curve is the measured data while the black curve is the fit curve produced using a 1:1 binding model. Instead, the SPR data provides qualitative support that MeBlu interacts with SARS-CoV-2 spike RBD.
